# Mitochondria and the Frozen Frog

**DOI:** 10.3390/antiox10040543

**Published:** 2021-04-01

**Authors:** Janet M. Storey, Shaobo Wu, Kenneth B. Storey

**Affiliations:** 1 Department of Biology, Carleton University, Ottawa, ON K1S 5B6, Canada; janstorey@cunet.carleton.ca; 2 Institute of Blood Transfusion, Chinese Academy of Medical Sciences (CAMS) and Peking Union Medical College (PUMC), Chengdu 610052, China; shaobowu123@126.com

**Keywords:** *Rana sylvatica*, antioxidant defenses, freezing survival, anoxia, dehydration, mitochondrial genes, *ATP 6/8*, *ND4*

## Abstract

The wood frog, *Rana sylvatica*, is the best-studied of a small group of amphibian species that survive whole body freezing during the winter months. These frogs endure the freezing of 65–70% of their total body water in extracellular ice masses. They have implemented multiple adaptations that manage ice formation, deal with freeze-induced ischemia/reperfusion stress, limit cell volume reduction with the production of small molecule cryoprotectants (glucose, urea) and adjust a wide variety of metabolic pathways for prolonged life in a frozen state. All organs, tissues, cells and intracellular organelles are affected by freeze/thaw and its consequences. This article explores mitochondria in the frozen frog with a focus on both the consequences of freezing (e.g., anoxia/ischemia, cell volume reduction) and mitigating defenses (e.g., antioxidants, chaperone proteins, upregulation of mitochondria-encoded genes, enzyme regulation, etc.) in order to identify adaptive strategies that defend and adapt mitochondria in animals that can be frozen for six months or more every year. A particular focus is placed on freeze-responsive genes in wood frogs that are encoded on the mitochondrial genome including *ATP6/8*, *ND4* and *16S* RNA. These were strongly up-regulated during whole body freezing (24 h at −2.5 °C) in the liver and brain but showed opposing responses to two component stresses: strong upregulation in response to anoxia but no response to dehydration stress. This indicates that freeze-responsive upregulation of mitochondria-encoded genes is triggered by declining oxygen and likely has an adaptive function in supporting cellular energetics under indeterminate lengths of whole body freezing.

## 1. Introduction

Every species on Earth is endowed with a set of biochemical and physiological strategies that can optimize their survival and reproductive capacity in the face of biotic and abiotic stresses. One of the most amazing survival strategies in the animal kingdom is freeze tolerance, the capacity of numerous invertebrate and a few vertebrate species to withstand ice formation within extracellular fluid spaces of their bodies while resisting intracellular freezing. This is a crucial adaptation for winter survival of many terrestrial ectotherms living in seasonally cold climates [1,2]. Freeze tolerance in nature can involve continuous freezing episodes lasting days, weeks or months. Some species (mainly insects) that overwinter in exposed sites above the snowpack may also experience daily freeze/thaw cycles. While frozen, normal physiological functions of tissues and organs are suspended including heartbeat, breathing, nerve transmission, skeletal muscle movement, etc.

Freeze/thaw is an event comparable to ischemia/reperfusion. Ice formation in extracellular and extra-organ spaces cuts off oxygen delivery to tissues. Hence, cells must use “survival mode” strategies including a switch to fermentable fuels (e.g., glucose, glycogen), accumulation of lactate as an end product, endurance of a slow but steady decrease in ATP levels and energy charge, and a necessary suppression of many ATP-expensive cellular activities [1,3]. As in other ischemic events (e.g., heart attack, stroke) damage due to increased reactive oxygen species (ROS) generation can be high during thawing when breathing and heartbeat resume and tissues undergo reperfusion and renewed oxygen uptake [4,5]. Hence, antioxidant defenses are proving to be an important feature of freeze tolerance.

Nowhere else in the cell is the effect of oxygen limitation felt more keenly than in the mitochondria, where the crucial function of the organelle in oxygen-dependent ATP production is suppressed or fully shut down in the frozen state with potentially grave consequences for the multitude of ATP-requiring cellular functions. Furthermore, freeze-induced disruption of mitochondrial functions can potentially trigger apoptosis. The present article explores the role of antioxidants and the adaptive responses of mitochondria to whole body freezing, summarizing research carried out on the primary vertebrate model of freeze tolerance, the wood frog, *Rana sylvatica*.

## 2. Characteristics of Freeze Tolerance

Among vertebrates, freeze tolerance is most common among amphibians that live at high latitudes or altitudes. Several species of frogs and two Siberian salamander species are known to be freeze tolerant and capable of surviving days, weeks or months of continuous freezing [1]. Hatchlings of a few turtle species that spend their first winter on land, typically buried in the natal nest, are also freeze tolerant, although they hibernate under water in subsequent winters. Among adult reptiles, freeze tolerance has been described for box turtles (*Terrepene carolina*) and European common lizards (*Lacerta vivipara*) [1].

The wood frog, *Rana sylvatica* (recently renamed *Lithobates sylvaticus*), is the best-studied of the freeze tolerant vertebrates and the main model used to explore molecular, biochemical and physiological mechanisms of freezing survival [1,2]. This North American frog has a range extending from the Appalachian mountains of southeastern USA, northward across all of the boreal forest of Canada and into Alaska. When frozen, wood frogs have no detectable vital signs: no heartbeat, breathing, blood circulation, muscle movement, or detectable brain activity. They survive for weeks/months with up to 65–70% of total body water converted into extracellular ice, much of the water drawn out of cells so that cell and organ volumes decrease dramatically. Hence, the frozen frog must deal with multiple freeze-induced consequences including (a) potential physical damage by ice crystals to delicate tissues, (b) major water loss from cells into extracellular or extra-organ ice masses, (c) hyperglycemia due to the extreme amounts of glucose mobilized to provide cryoprotection to cells (blood and tissue glucose levels can rise as much as 50-fold), and (d) anoxia/ischemia caused by the cessation of breathing, heartbeat and blood circulation [1]. Species that have evolved freeze tolerance appear to have done so because they are unable to access frost-free refugia such as by digging deep underground or moving into deep water. Among poikilothermic vertebrates freezing is usually triggered by skin contact with environmental ice when ambient temperature falls below the freezing point of body fluids (about −2 °C). Ice nucleation at mild subzero temperatures is actually beneficial for it allows a relatively slow progression of ice formation through extracellular fluid spaces, often requiring 15–20 h to reach maximum. Wood frogs use multiple protective measures that aid freezing survival including (a) synthesis of ice nucleating proteins (INPs) that help to modulate ice growth in extracellular spaces, (b) production of massive amounts of glucose as a cryoprotectant by liver that is then exported to all other organs and taken up to provide colligative resistance to the loss of cell water into extracellular ice, thereby retaining a minimum cell volume, (c) metabolic rate depression to slow energy use and suppress nonessential processes, (d) differential gene/protein expression to help ameliorate various consequences of life in a frozen state, and (e) altered regulation of the activities and properties of multiple enzymes [1,2]. 

## 3. Freeze Tolerance and Antioxidant Defenses

Studies of freeze tolerant animals can reveal information about how mitochondria respond to the very long interruptions of oxygen supply and the readjustments needed to deal with days, weeks or months of life in a frozen state. One biochemical adaptation that we have identified is the maintenance and/or upregulation of antioxidant defenses [6]. For example, freezing for 24 h at −2.5 °C strongly elevated total glutathione peroxidase activities in all five tissues of wood frogs tested (liver, kidney, brain, heart, skeletal muscle); activity in the liver rose by 50% and in the heart by 150% and remained high after thawing. In addition, glutathione peroxidase activities were significantly higher (often by ~2-fold) in *R. sylvatica* tissues as compared with a comparable species that winters under water in the same locale, the leopard frog *Rana pipiens*. The same was true of superoxide dismutase, glutathione *S*-transferase and glutathione reductase; enzyme activities in wood frog tissues were generally about twice as high as in leopard frogs [6]. Furthermore, glutathione contents (GSH, GSSG) in most tissues were markedly higher in frozen wood frogs than in unfrozen controls and also greatly exceeded the levels in *R. pipiens* tissues [6,7]. These responses by glutathione and antioxidant enzymes to freezing fit with the theory of “preparation for oxidative stress” (POS) whereby organisms may enhance antioxidant defenses in advance of a predictable seasonal or stress event (e.g., freeze/thaw, torpor/arousal, anoxia/reoxygenation) even though oxidative stress is a characteristic of the recovery phase when tissue oxygenation rises rapidly again [4].

Another study assessed changes in protein levels of five antioxidant enzymes (using a 5-plex Luminex Oxidative Stress Magnetic Bead panel) to compare responses to three stresses (freezing, anoxia, dehydration) in the brain and heart of *R. sylvatica* [8]. Note that anoxia and cell dehydration are two major component stresses of whole body freezing due to the cessation of blood circulation and the loss of much cell water into extracellular ice masses. In response to freezing, protein levels of cytosolic Cu/Zn superoxide dismutase (SOD) rose ~1.4-fold in the brain of frozen frogs, whereas the mitochondrial Mn-dependent SOD increased by the same amount in the heart [8]. Catalase protein levels in the brain were also strongly upregulated by 3-fold under anoxia stress but were unchanged in the heart. 

Similar context-specific patterns were found using a 5-plex Heat Shock Protein panel. Of particular interest, was the upregulation of Hsp60 (a mitochondrial HSP) in anoxic brain by 1.7-fold in response to 4 or 24 h of anoxia and by 2.1-fold in the heart of 4 h frozen frogs but with very little change in cytosolic HSPs [8]. Comparable data were gathered from an immunoblotting analysis of the responses of five chaperone proteins (Hsp110, Hsc70, Hsp60, Hsp40, Hsp10) in six tissues of wood frogs when faced with freezing, anoxia or dehydration stresses [9]. The two mitochondrial chaperones (Hsp60 and its partner Hsp10) were both significantly up-regulated by 1.5–2.3 fold in the liver, skin, kidney and skeletal muscle of wood frogs during freezing with singular up-regulation of Hsp60 in the brain and Hsp10 in the heart. Hsp60 was also up-regulated under anoxia stress (simulating the ischemia caused by freezing) in five tissues and under dehydration stress (simlulating water loss into extracellular ice masses) in three tissues with similar responses by Hsp10 [9]. Overall, HSP responses to stress by liver, brain and skeletal muscle were highest for the mitochondrial chaperones. Hence, this suggests a particular need to protect mitochondria under stress conditions with both enhanced antioxidant defenses and chaperones to stabilize protein structure. 

The responses of NF-κB, an oxygen-sensitive transcription factor, to wood frog freezing also support the proposal that enhanced antioxidant defenses contribute to cell protection in the frozen state. Under normal unstressed conditions, NF-κB is held in the cytoplasm by its inhibitory protein, IκB, but when ROS levels rise, IκB is phosphorylated, ubiquitinated and targeted for degradation, leaving NF-κB free to translocate into the nucleus and upregulate antioxidant genes [10]. Protein expression of both the p50 and p65 subunits of NF-κB was up-regulated by 2–2.7 fold in the liver and skeletal muscle of wood frogs after 4 and/or 24 h of freezing at −2.5 °C [11]. Phospho-IκB levels also rose in the liver indicating greater transcriptional activity of NF-κB and two proteins under NF-κB transcriptional control, ferritin heavy chain and manganese superoxide dismutase (MnSOD), showed 1.5–2-fold increases in the liver and skeletal muscle in the frozen state. Transcript levels of ferritin heavy chain were also elevated in the liver of 24 h frozen frogs but did not change in muscle. MnSOD is the isozyme found in mitochondria and its upregulation would supply enhanced protection from superoxide radicals generated in the shrunken cells of frozen animals. Freeze-triggered changes in antioxidants under NF-κB control would also enhance cellular antioxidant potential in anticipation of high oxidative stress when tissue perfusion restarts during thawing.

Upregulation of antioxidant defenses is also crucial for endurance of environmental stress in turtles to deal with anoxia/hypoxia stress in adult turtles during diving or underwater hibernation [12] or freezing stress among hatchling turtles that spend their first winter of life in their natal underground nests [13]. NF-κB responded to oxygen limitation in anoxia-tolerant adult red-eared slider turtles (*Trachemys scripta elegans*) with upregulation of both transcript and protein levels of the p50 and p65 subunits. The response was particularly strong after 5 h of anoxic submergence but returned to near baseline after 20 h submergence [12]. DNA-binding activity of NF-κB also increased by ~2-fold in the liver after 5 h anoxic submergence and remained high after 20 h anoxia. Downstream targets of NF-κB action (ferritin heavy chain, MnSOD, Cu/Zn SOD) were all significantly elevated by 1.5–2.5 fold after both 5 h and 20 h anoxia exposures [12]. MnSOD showed the largest increase in transcript levels (2.5 fold within 5 h), further indicating a crucial need to protect mitochondria under oxygen-limiting conditions. 

The action of another transcription factor involved in antioxidant defense, Nrf2, responded to whole body freezing in hatchling painted turtles (*Chrysemys picta marginata*). Both transcript and protein levels of Nrf2 were elevated in several tissues after 5 h of freezing of hatchlings and six isozymes of glutathione *S*-transferase (GSTP1, GSTM1, GSTM3, GSTK1, GSTA3 and GSTT1) increased significantly (by 1.5–2.5 fold) in selected tissues, primarily in the brain [13]. However, neither freeze/thaw nor anoxia/reoxygenation exposures of wood frogs had a substantial effect on Nrf2 levels in frog liver or skeletal muscle but Nrf2 binding capacity to DNA increased significantly in both tissues of 24 h frozen frogs as compared with unfrozen or 8 h thawed frogs [14]. Furthermore, protein levels of the MafG binding partner of Nrf2 rose strongly in response to 24 h anoxia in wood frog liver (by 2.5-fold) and muscle (by 6-fold) and remained high after 4 h aerobic recovery [15]. Both of these responses point to crucial roles for MafG and DNA binding capacity in mediating wood frog responses to Nrf2 under freezing or anoxia stresses. Indeed, Nrf2 belongs to the Cap ‘n’ Collar family of transcription factors that are unable to bind DNA on their own and, hence, dimerization with MAF proteins is obligatory in order to activate gene transcription [16]. This places stress-responsive changes in MafG levels as central to mediating changes in the expression of genes under Nrf2 control. 

Adaptation of antioxidant defenses in wood frogs was also analyzed at the gene transcript level. Multiple genes involved in antioxidant defense were identified as up-regulated in wood frog heart in response to freezing via cDNA array screening with human 19K cDNA gene chips [17]. These included several glutathione-S-transferase isozymes, thioredoxin, and glucose-6-phosphate dehydrogenase (the latter being key to producing the NADPH needed to recharge glutathione or thioredoxin). Genes coding for ferritin light chain and metallothionein 1G were also up-regulated and, together with ferritin heavy chain (discussed above), increased levels of ferritin and metallothionein can act to minimize metal-catalyzed oxidative stress in a situation where intracellular ion concentrations are strongly elevated due to water exit from cells to join extracellular ice masses. Screening also indicated upregulation of the *F*_o_ subunit C of the mitochondrial ATP synthase *F*_o_–*F*_1_ complex as well as the mitochondrial adenine nucleotide translocator, these changes implicating a need to readjust ATP production and ATP/ADP transport within cells in the frozen state [17].

## 4. Freeze Tolerance and Regulation of Antioxidant Enzymes

Not only are the activities of various antioxidant enzymes increased in wood frog tissues in response to freezing but enzymatic properties can also be modified by posttranslational modifications. For example, skeletal muscle catalase from frozen (−2.5 °C) wood frogs showed a significantly higher V_max_ (1.5-fold) and lower K_m_ H_2_O_2_ (0.64 fold) compared with catalase from control (5 °C acclimated) frogs [18]. These changes were also seen during in vitro incubations that stimulated protein kinase A (PKA) or the AMP-activated protein kinase (AMPK) whereas protein phosphatase treatment increased the K_m_ value. Properties of both cytoplasmic Cu/Zn-dependent superoxide dismutase (CuZn-SOD) and mitochondrial Mn-dependent SOD (MnSOD) also changed in wood frog skeletal muscle during freezing [19]. Cu/Zn SOD showed an increased V_max_ in muscle from frozen frogs but no significant change in kinetic parameters whereas MnSOD from muscle of frozen frogs showed increased affinity for substrate and reduced sensitivity to urea denaturation, as compared with controls. Total phosphorylation of MnSOD also increased ~2.8-fold for the muscle enzyme from 24 h frozen frogs, comprised of 2.4- and 1.3-fold increases in serine and tyrosine phosphorylation of the protein. This indicates that substantial posttranslational modifications can stabilize/adapt MnSOD to deal with an altered intramitochondrial environment in the frozen state. 

Both superoxide radicals and hydroxyl radicals (a product of H_2_O_2_ breakdown) have damaging effects on cell proteins and membrane phospholipids. However, H_2_O_2_ also acts as a signaling molecule that is exported from mitochondria to trigger and coordinate responses by a variety of extra-mitochondrial cell functions, both protective and destructive [20]. Hence, mitochondria are now considered as both receivers and integrators of diverse cell signals, an action that could certainly be important for cell/organ survival in freeze tolerant animals whose cells are cut-off from global signals (hormones, nerve stimulation) throughout the freeze. Freeze tolerant animals may need to revert to dependence on only ancient single cell mechanisms for maintaining homeostasis throughout what could be days or months of living in a frozen state. 

## 5. Freeze-Induced Expression of Genes/Proteins with Mitochondrial Functions 

Freezing triggers the upregulation of many genes in wood frog organs whose protein products work in multiple capacities to reorganize metabolism or protect cells/tissues from a variety of issues. Various studies have identified genes and their protein products that function in modulating where ice grows, mediating cryoprotectant synthesis and its export/uptake by cells, coordinating metabolic rate suppression, defending cells from ischemia/reperfusion injury, stabilizing membranes as cell volumes fall to low levels, and enhancing clotting factors to deal with any bleeding issues after thawing, among others [1,6,9,21,22,23]. A number of these address mitochondrial concerns.

Several freeze-upregulated genes code for proteins that function within mitochondria or are genes that are coded on the mitochondrial genome. An example of the former is the adenine nucleotide translocator (ANT; also called ADP/ATP translocase) that is a highly abundant protein located in the inner mitochondrial membrane. Screening of a cDNA library made from liver of freeze-exposed (−2.5 °C for 24 h) versus control (5 °C-acclimated) wood frogs confirmed ANT as freeze-upregulated [24]. ANT catalyzes adenylate exchange, moving newly synthesized ATP out of mitochondria in exchange for ADP import from the cytoplasm. Transcript levels of *aat* rose 4.5-fold in the liver of wood frogs within 8 h of freeze initiation and protein levels peaked at 24 h frozen. Furthermore, *aat* transcript levels in wood frog tissues also responded to anoxia exposure, one of the component stresses of freezing, but did not respond to dehydration [24]. This indicated that freeze-induced ANT upregulation is linked to cellular energetics and responding to the effect of oxygen limitation in compromising tissue ATP supply. Complementing *aat* upregulation, both gene and protein expression of the inner mitochondrial membrane inorganic phosphate carrier (PiC), that supplies the P_i_ needed for ATP synthesis, were strongly upregulated by freezing in wood frog liver [25]. Overall, these adjustments to inner mitochondrial membrane function suggest that mitochondrial ATP production is supported for as long as possible even as oxygen delivery to tissues is increasingly restricted by rising body ice content and reduced blood circulation. Such protective actions can support the crucial role of the liver in producing the glucose cryoprotectant that is synthesized and exported to all other organs during the early minutes/hours after freezing begins. Levels of the mitochondrial chaperone protein, HSP60, that functions to fold or refold proteins in the mitochondria also rose by 1.5–2.2-fold in most wood frog tissues during freezing indicating that attention is also given to stabilizing proteins in mitochondria in order to maintain protein structure/function as freezing progresses [9]. HSP60 in wood frog liver also responded positively to both anoxia and dehydration stresses confirming its importance in stabilizing mitochondrial proteins in response to multiple external stresses.

Changes in the proteome of wood frog liver between summer and winter have also been identified using liquid chromatography coupled with tandem mass spectrometry quantitative isobaric peptide mapping [26]. Proteins that were more abundant in winter included some involved in glycogen metabolism (phosphorylase, protein phosphatase-1), glycolysis (pyruvate kinase, glyceraldehyde-3-P dehydrogenase), heat shock proteins, and the antioxidant peroxiredoxin 6 (PRDX6). Importantly, PRDX6 is known to translocate into mitochondria and does so as a response to ischemia/reperfusion stress or reactive oxygen species generation [27,28,29]. Within the mitochondria PRDX6 is involved in controlling oxidative stress with respect to PINK1/Parkin-mediated mitophagy [28]. Hence, upregulation of PRDX6 provides another indication that wood frog cells actively enhance antioxidant defenses, particularly within the mitochondria, to protect themselves during the ischemia/reperfusion events of natural freeze/thaw. DNA array screening also revealed freeze-responsive changes in gene expression in the liver and heart of freeze-tolerant hatchling painted turtles, *C. picata marginata*. Upregulation of several proteins associated with antioxidant defense (by 1.6- to 3.5-fold) was identified for iron binding proteins (ferritin heavy and light chains, and transferrin receptor) and antioxidant enzymes (glutathione peroxidase, glutathione-*S*-transferase, peroxiredoxin) [13], further supporting the proposal that strong antioxidant defenses are key to freeze tolerance.

## 6. Enzymatic Controls on Mitochondrial Metabolism in Frozen Frogs

The above sections dealt mainly with measures that can protect or stabilize mitochondria during freezing in wood frogs. However, adaptive regulation of the activities of mitochondrial enzymes also deserve consideration. Although oxygen depletion will ultimately halt or strongly restrict oxygen-dependent mitochondrial functions (e.g., electron transport chain, oxidative phosphorylation, and beta-oxidation of fatty acids), regulatory controls on selected enzymes could still mediate or mitigate other metabolic functions of mitochondria.

For example, various aspects of amino acid metabolism continue in the frozen frog. Cellular energetics are supported by anaerobic metabolism in frozen frogs with the accumulation of both L-lactate and L-alanine as glycolytic end-products [3,30]. Alanine production from pyruvate reduces the acidic consequences of lactic acid accumulation, an advantage in a closed system such as frozen tissues. Indeed, alanine accumulation in wood frog tissues (particularly skeletal muscle) was inversely related to decreases in aspartate, glutamate and glutamine levels [3], these three amino acids being readily able to participate in transaminase or deaminase reactions to produce alanine.

Wood frogs also build up urea in tissues during freezing and this small molecule supplements glucose as a colligative cryoprotectant. Urea is a known osmoprotectant in amphibians that undergo dehydration stress. In particular, numerous frogs and toad species enter a hypometabolic state called estivation in the hot dry summer months. During this time they undergo progressive muscle atrophy as they catabolize muscle protein for fuel while utilizing the amino groups released to produce urea that is retained as a colligative deterrent to water loss [31]. Hence, it is not surprising that urea accumulation is also triggered in amphibian cells undergoing dehydration as a consequence of cell water loss into extracellular ice masses [32,33].

A key player in amino acid catabolism and urea synthesis is glutamate dehydrogenase (GDH), its reversible reaction allowing GDH to act as both a nitrogen donor and acceptor. Catabolism of several amino acids (glutamate, glutamine, histidine, arginine, proline) ends via GDH with the product, alpha-ketoglutarate (α-KG), funneled into the Krebs cycle and the ammonium ion either excreted or used for urea synthesis. An analysis of purified wood frog liver GDH showed multiple properties that could promote amino catabolism in support of urea synthesis. For example, when assayed at 22 °C in the direction of glutamate catabolism (glutamate + NAD^+^ → α-KG + NH_4_^+^ + NADH + H^+^), the liver enzyme from frozen frogs, compared with controls, showed a 39% higher activity of GDH. K_m_ values for glutamate and NAD^+^ were both ~60% lower (indicating greater affinity for these substrates) and inhibition by GTP was greatly reduced [34]. Hence, the enzyme from frozen liver appeared poised for amino acid catabolism and for NH_4_^+^ production to feed urea synthesis. GDH from liver of control versus frozen frogs also differed in posttranslational modifications with the enzyme from liver of frozen frogs showing reduced acetylation and ADP-ribosylation but elevated lysine methylation as compared with controls [34]. These differential modifications could contribute to altering multiple features of GDH function or localization; for example, the maximum velocity of the enzyme in the glutamate catabolizing direction was 25% higher for liver GDH from frozen frogs when assayed at 5 °C as compared with the enzyme from control frogs.

Crucial first steps of the urea cycle also take place in the mitochondria catalyzed by carbamoyl phosphate synthetase 1 (CPS1) and ornithine transcarbamylase (OTC). Differential regulation of CPS1, the rate-limiting enzyme of urea synthesis, was detected in the liver of control (5 °C) versus frozen (−2.5 °C) wood frogs [35]. This enzyme catalyzes the first step of the cycle by trapping NH_4_^+^ in an ATP-driven ligation with bicarbonate and phosphate to form carbamoyl phosphate (CP). CP is then attached to ornithine via OTC to form citrulline and exported to the cytoplasm where the remaining reactions of cycle are completed, ending in urea output. Purified CPS1 from liver of frozen versus control frogs showed significant changes in properties including a higher maximal activity, greater affinity for NH_4_^+^, reduced sensitivity to urea inhibition and, in the presence of high glucose, greater affinity for ATP and the obligate CPS1 activator, *N*-acetylglutamate. CPS1 from control and frozen frogs also differed in posttranslational modification by lysine glutarylation that was significantly reduced on the enzyme from frozen frogs [35]. These results indicate that urea cycle activity is maintained and differentially regulated during freezing and that CPS1 activation during freezing has an important contribution to make to cryoprotection by increasing the osmotic resistance (via facilitating urea accumulation) against cell water loss into extracellular ice. These enzymatic studies show that, despite the frequent focus on cellular energetics as the crucial role of mitochondria, these organelles have other functions to contribute to achieve biochemical adaption to environmental stress.

## 7. The Mitogenome of Freeze Tolerant Frogs

**Figure 1:**
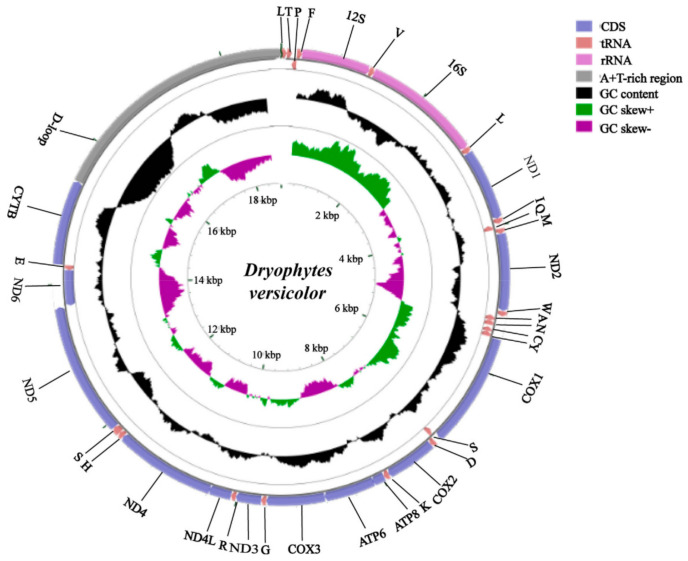
The mitochondrial genome of the freeze tolerant North American gray treefrog, *Dryophytes versicolor* (formerly *Hyla versicolor*). The outer ring shows the gene map with protein coding genes, rRNAs, tRNAs and the AT-rich region. Genes seen on the outer ring are coded on the heavy (majority) strand whereas genes on the inner ring are coded on the light (minority) strand. Other circles show GC content and GC skew as shown in the color-coded legend. From [38].

The mitochondrial genome is a small closed circular molecule (typically about 16,000 bp) and contains 13 protein-coding genes (*ATP6/8*, *CytB*, *COX1-3*, *ND1-6* and *ND-4L*), 22 tRNAs, 2 rRNA genes and a noncoding control region [36]. It is widely used as a tool to assess phylogenetic relationships between species due to its simplicity, maternal inheritance, and high evolutionary rate. The wood frog mitochondrial genome has been sequenced and shows the typical vertebrate content (GenBank accession number KP222281) [37]. No unusual features were noted and the genome sequence was almost identical to that of the North American bullfrog, *Lithobates catesbeianus* (that winters underwater), and three ranid species found in northeastern Asia. The mitochondrial genome of another freeze tolerant frog, the North American treefrog *Hyla versicolor* (recently renamed *Dryophytes versicolor*) has also been sequenced and Figure 1 shows the genome map of this species that is typical of vertebrates [38].

## 8. Freeze Responsive Expression of Wood Frog Mitochondria-Encoded Genes

A search for mitochondria-encoded genes that were freeze-responsive in wood frog tissues revealed up-regulation of several genes. Screening of a wood frog brain cDNA library via Northern blotting using ^32^P-labeled cDNA probes and poly(A)^+^ mRNA from brain of control frogs (5 °C acclimated) vs. frozen frogs (−2.5 °C, 24 h) revealed enhanced expression of *ATP synthase 6/8* and *16S rRNA* genes during freezing [39]. In addition, screening of a liver cDNA library highlighted up-regulation of the NADH-ubiquinone oxidoreductase subunit 4 gene (*ND4*) [39].

Several nuclear-encoded genes were also identified as up-regulated from these same screens including ribosomal protein L7, acidic ribosomal phosphoprotein P0, phosphoglycerate kinase 1 (PGK1), and a novel protein that was named Li16 [40,41,42,43,44]. These have all proven to play valuable roles in freezing survival. P0 is part of a 5-protein ribosomal complex P0(P1-P2)_2_ and upregulation of P0, enhancing complex formation, can exert inhibitory control on ribosome assembly under low oxygen conditions [45]. Hence, in wood frogs, freeze-responsive upregulation of P0 can potentially contribute to energy savings by limiting protein translation in the anoxic frozen state. PGK1 is one of two glycolytic enzymes that produce ATP. Its expression is under the control of the hypoxia-inducible transcription factor-1 [46]. PGK1 up-regulation during freezing can lead to enhanced anaerobic ATP production in the frozen state, as evidenced by the accumulation of lactate and alanine as glycolytic end products in organs of frozen frogs [3,30]. Studies of Li16 and another freeze responsive novel protein identified earlier, FR10 [44], indicate that these can interact with the plasma membrane on the inner versus outer sides, respectively [42]. Their action can potentially help to stabilize membranes as cell volume shrinks due to water exit into extracellular ice crystals. A recent study also showed the ability of FR10 to inhibit ice recrystallization, suggesting that it also acts to minimize physical damage by extracellular ice crystals [23], a potentially critical action to minimize ice damage to delicate organs or capillaries. Whether Li16 or other proteins also act to stabilize membranes of intracellular organelles during cell volume changes associated with freeze/thaw is still unknown.

**Figure 2:**
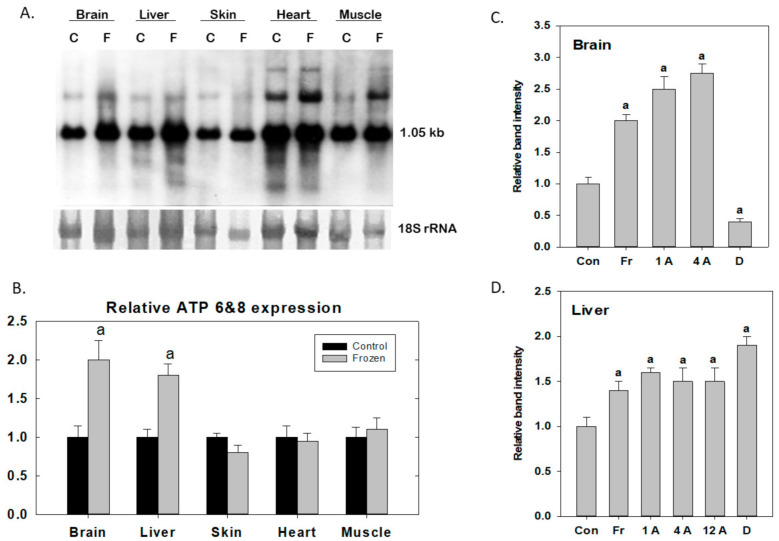
Expression of mitochondrial ATP 6/8 genes under freezing, anoxia and dehydration stresses in wood frog tissues, as assessed by Northern blotting. (**A**). Equal amounts of total RNA (15 μg) were loaded onto formaldehyde agarose gels followed by electrophoresis for 1.5 h at 80 V. The ATP6/8 band was detected at ~1.05 kb and band densities were quantified (using Imagequant v3.22) and standardized against densities of corresponding 18S rRNA bands (detected by ethidium bromide staining). Labels are: C: samples from control, cold-acclimated frogs (5 °C for 2 weeks), and F: cold-acclimated frogs subjected to freezing at −2.5 °C for 24 h. (**B**). Histogram showing mean band densities ± SEM for *n* = 3–5 Northern blots from control vs. frozen frogs; ^a^ -significantly different from the corresponding control, *p* < 0.05. (**C**,**D**). Histograms showing mean band densities ± SEM for *n* = 3 determinations of ATP 6/8 expression under control (Con), 24 h frozen (Fr), anoxia exposure for 1, 4 or 12 h at 5 °C (**A**), and dehydration to 40% of total body water lost at 5 °C (D). For more details of experimental procedures, see [39,40,41].

Freeze-upregulated mitochondria-encoded genes of *R. sylvatica* were also identified with interesting results. The mitogenome encodes two subunits (ATP synthase 6 and 8) of the mitochondrial F_o_ complex (the transmembrane proton channel of the inner mitochondrial membrane) that is part of the F_o_F_1_ ATP synthase, also known as complex V of the electron transport chain. These two genes share an unusual characteristic, a 46 nucleotide overlap of their sequences, with *ATP8* being coded via the +1 reading frame whereas *ATP6* gene is read on the +3 reading frame. Hence, the genes are typically referred to as *ATP 6/8*. The *ATP 6/8* transcript retrieved from a wood frog brain cDNA library (clone Br3, RNA size ~1.05 kb) [39] showed ~2-fold up-regulation in response freezing exposure (24 h at −2.5 °C) in both brain and liver, as assessed by Northern blotting, in comparison with control frogs held at 5 °C (Figure 2A,B). However, *ATP 6/8* transcript levels were unaffected in skin, heart and skeletal muscle.

*ATP 6/8* expression was also independently assessed in tissues from wood frogs exposed to two component stresses of freezing: anoxia (frogs given 24 h under a nitrogen gas atmosphere at 5 °C) and dehydration (frogs held at 5 °C in dry containers until evaporative loss of 40% of total body water was reached). Both of these individual stresses are fully survivable by wood frogs [1]. Transcript levels of *ATP 6/8* responded robustly to anoxia rising within 1 h by ~2.5-fold in the brain (Figure 2C) and ~1.5 fold in the liver (Figure 2D) and remaining high over time. However, the response to whole body dehydration was organ-specific, brain showing suppressed levels of *ATP 6/8* transcripts (reduced to ~40% of controls; Figure 2C) whereas liver transcript levels rose to ~1.9-fold over controls (Figure 2D). These data suggest that the impact of freezing on mitochondria-encoded gene expression is largely a response to oxygen restriction (not cell volume reduction). This is not surprising given the central role of mitochondria in oxygen-dependent ATP synthesis. Responses to dehydration were tissue-specific and in the brain may reflect metabolic rate depression to reduce energy demand. By contrast, the liver has a major metabolic role to play under dehydrating conditions, since the wood frog liver also synthesizes and exports high quantities of glucose and urea as osmoprotectants (comparable to the response to freezing) to help sustain viable cell volumes as well as retard evaporative water loss across the skin [1]. Hence, the metabolic response to dehydration in the liver (that occurs under aerobic conditions) appears to include upregulation of mitochondrial electron transport and ATP production capacity.

The mitochondria-encoded gene *ND4* (NADH-ubiquinone oxidoreductase, subunit 4) was also upregulated by freezing as determined by screening a liver cDNA library. ND4 protein is one of the seven subunits of NADH-ubiquinone oxidoreductase (complex 1 of the electron transport chain) that are encoded on the mitochondrial genome, all of which are hydrophobic subunits found in the transmembrane portion of the large 44 subunit ND protein. A single band was detected on a RNA blot at ~1.4 kb when loaded with liver total RNA samples and probed with liver clone Li39s insert [39]. Transcript levels in the liver increased by 1.7 ± 0.1-fold after 4 h freezing at −2.5 °C and stabilized at 1.5-fold over controls after 12 or 24 h frozen (as compared with 5 °C controls) before returning to control values after thawing. Liver *ND4* transcripts also increased strongly when frogs were under anoxia exposure but decreased in frogs dehydrated to 40% of total body water lost. Once again, this gives evidence that freeze-induced upregulation of a gene on the mitochondrial genome is a response to oxygen restriction.

The mitochondrial genome is widely used by researchers investigating phylogenetic relationships among species but equally there is interest in determining if changes in the mitogenome aid animals in adapting to environmental pressures that could, in some cases, ultimately define a new species. For example, for birds living at or flying over high altitude locations, hypoxia is unavoidable and yet high rates of aerobic ATP production are needed to power flight. As such, every step in aerobic respiration could undergo selection to increase efficiency as illustrated for cytochrome c oxidase by Scott et al. [47] in studies of bar-headed geese that fly as high as 9000 m to migrate over the Himalayas. Two recent studies of adaptation to high altitude (typically characterized by both hypoxia and low temperatures) provide further insights. Zhou et al. [48] found positive selection of *ND2, ND4* and *ATP6* genes in high altitude lineages (>3000 m) of galliform birds as compared with related lowland species. Similarly, Jin et al. [49] reported positive selection of *ND2, ND3, ND4, ND5* and *ND6* among different Chinese toad-headed lizard species of the genus *Phrynocephalus* that range over a 4200 m altitudinal gradient. Both of these studies support the idea that small changes in the sequences of mitochondria-encoded proteins (and their expression) could have a crucial impact on hypoxia tolerance and cold hardiness among various amphibian and reptile species living in seasonally cold environments, including those that use strategies of freeze tolerance. This is certainly an idea worth further exploration.

The mitochondrial genome also encodes 16S and 12S ribosomal RNA genes (Figure 1) that, together with ribosomal proteins, form the ribosomes that translate the 13 protein-coding genes of the mitogenome, all of which are subunits of the electron transport complexes found in the inner mitochondrial membrane. The mitochondrial *16S RNA* gene was identified as upregulated in selected tissues of freeze-exposed wood frogs as determined by Northern blotting [39]. A strong band at ~1.6 kb was detected when probed with a brain clone (Br4) and after 24 h freezing at -2.5 °C, 16S rRNA transcript levels had risen significantly by 1.6 ± 0.10-fold in the brain, 1.5 ± 0.05-fold in the liver and 2.0 ± 0.04-fold in skeletal muscle, as compared to 5 °C controls. Transcripts remained high in the brain and muscle after 24 h thawing at 5 °C whereas 16S rRNA levels returned to control levels in the liver. Levels of 16S RNA in wood frog brain also responded robustly to 4 h of anoxia exposure, rising by 4.8 ± 0.2-fold but were unaffected by dehydration stress. Hence, freezing upregulates not only a range of mitochondrial encoded proteins but also triggers the production of crucial rRNA components of mitochondrial ribosomes in order to support the intramitochondrial synthesis of key proteins of the electron transport chain.

## 9. Mitochondrial Genes and the Freeze-Tolerant Gray Tree Frog

Considerable research has also been done on the biochemical adaptations to freezing used by North American gray tree frogs, both the tetraploid *Hyla versicolor* (recently renamed *Dryophytes versicolor*) and the closely related Cope’s gray treefrog (*D. chrysoscelis*) (diploid) [1]. Both use glycerol as their cryoprotectant and *D. chrysoscelis* has been used for extensive studies of glycerol import/export from cells and analysis of the aquaglyceroporin (a modified aquaporin) that conducts this transport [50].

A study of the mitochondrial genome of *H. versicolor* (Figure 1) has also provided information about the responses to stress by mitochondrial protein-coding genes [38] by evaluating the effects of freezing (−2.5 °C, 24 h) and anoxia (N_2_ atmosphere, 5 °C, 24 h) on their expression. Anoxia exposure led to a 2.4-fold increase in cytochrome c oxidase, subunit 2 (*COX2*) transcript levels in *H. versicolor* skeletal muscle whereas *ND3* transcripts were reduced by 50%. Freezing had somewhat different effects; *COX1* transcript levels were significantly reduced to ~60% of the control value and *COX2, ND3* and *ATP6* transcript levels also decreased by about 25–30%. The common factor in these stress responses was a reduction in *ND3* transcripts under both conditions, which might suggest a possible regulatory role for this subunit of the first respiratory chain complex that could help to modulate respiratory chain activity in response to oxygen availability.

## 10. Conclusions

Freeze tolerance is an amazing adaptation exhibited by only a few vertebrate species. Pre-existing well-developed tolerances of anoxia and/or dehydration are well known components of freeze tolerance. The present paper highlighted the major importance of antioxidant defenses to freezing survival and emphasized a new concept, the key role for mitochondria in freezing survival. This is supported by the upregulation of a variety of genes (on both nuclear and mitochondrial genomes) that code for proteins residing in mitochondria. These proteins can provide either protective actions or support continuing low level mitochondrial ATP production for as long as oxygen reserves permit.
